# Exploration of the potential common pathogenic mechanisms in COVID-19 and silicosis by using bioinformatics and system biology

**DOI:** 10.1007/s10142-023-01092-2

**Published:** 2023-06-06

**Authors:** Yunze Tian, Beibei Yu, Yongfeng Zhang, Sanpeng Zhang, Boqiang lv, Shouping Gong, Jianzhong Li

**Affiliations:** 1 Department of Thoracic Surgery, the Second Affiliated Hospital of Xi’an Jiao Tong University, Shaanxi Province, Xi’an, 710004 China; 2 Department of Neurosurgery, the Second Affiliated Hospital of Xi’an Jiao Tong University, Shaanxi Province, Xi’an, 710004 China; 3 Operating room, the Second Affiliated Hospital of Xi’an Jiao Tong University, Shaanxi Province, 710004 Xi’an, China

**Keywords:** COVID-19, Silicosis, Weighted correlation network analysis, ScRNA-seq

## Abstract

Silicosis is an occupational lung disease that is common worldwide. In recent years, coronavirus disease 2019 (COVID-19) has provided daunting challenges to public healthcare systems globally. Although multiple studies have shown a close link between COVID-19 and other respiratory diseases, the inter-relational mechanisms between COVID-19 and silicosis remain unclear. This study aimed to explore the shared molecular mechanisms and drug targets of COVID-19 and silicosis. Gene expression profiling identified four modules that were most closely associated with both diseases. Furthermore, we performed functional analysis and constructed a protein–protein interaction network. Seven hub genes (budding uninhibited by benzimidazoles 1 [BUB1], protein regulator of cytokinesis 1 [PRC1], kinesin family member C1 [KIFC1], ribonucleotide reductase regulatory subunit M2 [RRM2], cyclin-dependent kinase inhibitor 3 [CDKN3], Cyclin B2 [CCNB2], and minichromosome maintenance complex component 6 [MCM6]) were involved in the interaction between COVID-19 and silicosis. We investigated how diverse microRNAs and transcription factors regulate these seven genes. Subsequently, the correlation between the hub genes and infiltrating immune cells was explored. Further in-depth analyses were performed based on single-cell transcriptomic data from COVID-19, and the expression of hub-shared genes was characterized and located in multiple cell clusters. Finally, molecular docking results reveal small molecular compounds that may improve COVID-19 and silicosis. The current study reveals the common pathogenesis of COVID-19 and silicosis, which may provide a novel reference for further research.

**Supplementary Information:**

The online version contains supplementary material available at 10.1007/s10142-023-01092-2.

## Introduction

Coronavirus disease 2019 (COVID-19) is a highly infectious pneumonia caused by the severe acute respiratory syndrome coronavirus 2 (SARS-CoV-2) and has been regarded as a global pandemic (Sharma et al. 2021). As of 5:43 p.m. CEST on October 28, 2022, there has been 626,337,158 confirmed cases of COVID-19 globally, including 6,566,610 deaths reported to the World Health Organization.^1^After a COVID-19 infection, a few cases may progress to interstitial pneumonia, often leading to pulmonary fibrosis (George et al. 2020; McDonald 2021). With the ravaging effects of COVID-19, the comorbidities between COVID-19 and other respiratory diseases remain a major concern.

Silicosis is generally caused by long-term exposure to dust containing excessive amounts of silica or crystalline silica (Steenland and Ward 2014). Occupations such as mining or natural gas mining are more likely to expose workers to silica (Naidoo and Jeebhay 2021). Silica-induced silicosis is an occupational disease characterized by progressive lung fibrosis (García-Núñez et al. 2022; Leung et al. 2012). The incidence of silicosis remains high worldwide, especially in underprivileged communities (Leung et al. 2012). A previous study reported that more than 2.3 million workers worldwide are exposed to hazardous levels of silica nanoparticles (Mazurek et al. 2017).

Pulmonary fibrosis is a type of pulmonary interstitial disease that is harmful and difficult to cure. Once the patient is not properly treated after the onset of the disease, it is likely that life, health, and safety will be threatened in a short time (Savin et al. 2022). However, both COVID-19 and silicosis present the risk of developing pulmonary fibrosis, and patients with both diseases generally tend to be more seriously ill than those with either disease (Naidoo and Jeebhay 2021). The pathological basis of pulmonary fibrosis in COVID-19 is severe damage to the alveolar epithelium and massive infiltration and reorganization of inflammatory cells, which eventually lead to alveolar atrophy and parenchymal fibrosis (John et al. 2021). In addition, silicosis pulmonary fibrosis is characterized by disturbance of alveolar structure and diffuse inflammation. In the early stage of silicosis fibrosis, effector cells secrete many cytokines, induce macrophage alveolar inflammation, and form pulmonary fibrosis in the process of inflammatory injury and repair (Tan and Chen 2021). Although many cases of COVID-19 have been reported among patients with silicosis, the shared biological pathways involved in the two diseases are not fully understood. This has attracted the attention of the medical community.

With the development of transcriptome sequencing technology, researchers can rapidly detect the expression levels of large-sized genes in tissues, which is beneficial for a deeper understanding of disease pathogenesis. In our study, weighted correlation network analysis (WGCNA) was performed based on the COVID-19-related dataset (GSE157103) and silicosis-related datasets (GSE49144 and GSE32147) to identify shared gene clusters between the two diseases. This approach routinely identifies the biological processes shared by genes in two disease phenotypes (Sezin et al. 2018; Yao et al. 2021; Zhu et al. 2020). The co-expression modules in COVID-19 and silicosis were identified using bulk RNA sequencing (bulk RNA-seq). The gene set enrichment analysis was performed to explore the biological processes that may occur simultaneously in these two diseases. Subsequently, the hub-shared genes were screened in the protein–protein interaction (PPI) networks. Furthermore, transcription factor (TF)-gene interactions and gene-microRNA (miRNA) regulatory networks were constructed based on known hub-shared genes. In addition, based on the single-cell RNA sequencing (scRNA-seq) dataset GSE182123, the expression of hub-shared genes was characterized and located in multiple cell clusters. Finally, we performed a molecular docking study of potential drugs for hub-shared genes. This is the first study to identify shared genomic and molecular mechanisms between COVID-19 and silicosis.

## Materials and methods

### Dataset and source

**Fig. 1:**
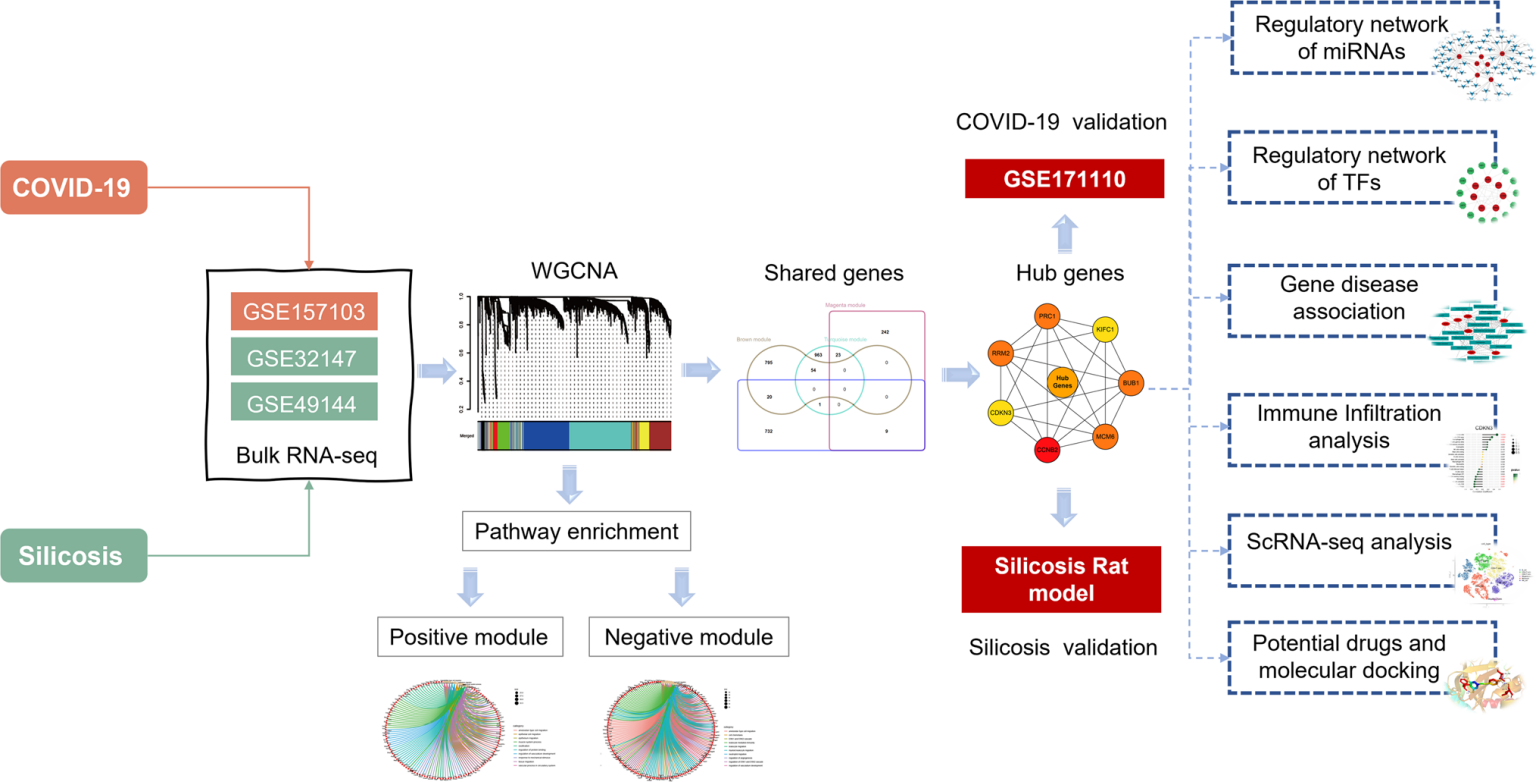
The flowchart of the current study. WGCNA: weighted gene co-expression network analysis; TFs: transcription factors; sc-RNA seq: single-cell RNA sequencing

The overall flowchart of this study is shown in Fig. 1. The Gene Expression Omnibus^2^ (GEO) database was used to download the gene expression profiles analyzed in this study. In bulk RNA-seq of silicosis, we extracted rat lung tissue sequencing data from GSE32147 and GSE49144 (Sellamuthu et al. 2013; Umbright et al. 2017). Subsequently, 22 rats were treated with 15 mg/m^3^ of crystalline silica for 2–4 weeks, and 14 control rats were used in the analysis matrix for silicosis. The GEO accession ID of COVID-19 was GSE157103, which included the bulk RNA-seq expression matrix from 128 human peripheral blood samples (102 COVID-19 samples and 26 healthy samples) (Overmyer et al. 2021). In addition, the dataset GSE171110, consisting of 10 healthy control peripheral blood samples and 44 COVID-19 samples, was utilized as a validation dataset in our study. Finally, we obtained peripheral blood scRNA-seq data from GSE182123 with four COVID-19 patients and four healthy people (Choi et al. 2022). The sample sizes of the datasets included in the study are shown in Supplementary Table 1.

### Establishment of a silicosis animal model

Male Sprague–Dawley rats weighing 200–250 g were used in this study. The rats were housed in a temperature-controlled room with a 12-h light/dark cycle and had free access to food and water. All animal experiments were approved by the Institutional Animal Care and Use Committee (Xi'an Jiaotong University). The silica inhalation method was used to prepare the silica-induced pulmonary fibrosis model. Briefly, six rats were randomly divided into two groups: the control group and the silica group. The rats in the silica group were placed in a chamber and exposed to silica particles (15 mg/m^3^) for four h per day, 5 days per week, for four weeks. The rats in the control group were placed in the same chamber and exposed to an equal volume of normal saline. To gain further insight into the reliability of the silicosis model in rats after a four-week exposure period, we sought to detect morphological changes by hematoxylin and eosin (HE) and Masson trichrome staining techniques. Two groups of rats were anesthetized with isoflurane under an anesthetic apparatus and then perfused with phosphate buffer to obtain lung tissue. Rat lung tissue specimens were prepared with 4% paraformaldehyde, fixed at 4 °C for 48 h, embedded in paraffin, and then sliced (slice thickness: 4 µm). HE and Masson trichrome staining observed pathological changes in the control and silicosis groups.

### Weighted gene co-expression network analysis

The R software (v4.1.2; R Foundation, Vienna, Austria) was used for all analyses and visualizations performed in this study. All raw matrices of bulk RNA-seq were combined with the RNA probes after log normalization to form the subsequent analysis profiling matrix. Batch effects were removed from the gene expression profiles by merging GSE32147 and GSE49144 using the surrogate variable analysis (sva) package in R. Furthermore, the WGCNA package was used to perform module analysis. After screening genes with a variance > 25%, the suitable power was determined using the pickSoftThreshold function. The genes with similar expression profiles were categorized into gene modules using the Therapy Outcome Measure (TOM)-based dissimilarity measure through average linkage hierarchical clustering. Finally, the correlation coefficients between each module and the sample trait in COVID-19 and silicosis were assessed. Four module genes with the strongest positive and negative correlations in COVID-19 and silicosis were selected for subsequent analysis.

### Protein interaction network and pathway enrichment analysis

Evenn (Chen et al. 2021),^3^a free online website, was used to screen shared genes involved in COVID-19 and silicosis. Metascape^4^ and Bioinformatics^5^ websites were used to find gene ontology (GO) enrichment pathways involved in shared genes. The protein–protein interaction network was exported from the Search Tool for the Retrieval of Interacting Genes/Proteins (STRING) database^6^ and optimized using Cytoscape software. Moreover, the Minimal Common Oncology Data Elements (mCODE) plugin was used to identify highly interconnected gene clusters in shared genes.

### Quantitative real-time polymerase chain reaction (RT-PCR)

At the end of the 4-week exposure period, the rats were euthanized, and their lungs were immediately stored in liquid nitrogen. Total RNA was extracted from tissues using the TRIzol reagent according to the manufacturer’s instructions (Invitrogen, USA). Reverse transcription (RT) was performed using the PrimeScript RT Reagent Kit (TaKaRa, Japan). The reaction conditions were as follows: 37 °C for 15 min, 85 °C for 5 s, and then held at 4 °C. The primer sequences of hub genes are listed in Supplementary Table 2. The relative gene expression levels were calculated using the 2^−ΔΔCt^ method. Glyceraldehyde-3-phosphate dehydrogenase (GAPDH) was used as an internal control. The data were expressed as mean ± standard deviation (SD). Statistical comparisons were performed using the Student’s *t*-test; differences with *P* < 0.05 were considered statistically significant.

### Immune infiltration analysis

We used the CIBERSORT algorithm^7^ to compute the samples’ immune cell abundance. The pheatmap package was used to describe the contents of the 22 types of immune cells in COVID-19. Spearman’s correlation analysis was then performed to detect correlations between immune cells and gene expression levels. Finally, a lollipop chart was created using the ggplot2 package to illustrate the relationship between hub-shared genes and immune cells.

### TFs, miRNAs, and the disease regulatory network

In the Enrichr database,^8^we used the JASPAR and TargetScan modules to predict the TFs and miRNAs that may be combined with hub-shared genes. The DisGeNET^9^ database was used to predict the diseases related to hub-shared genes. The TFs, miRNAs, and disease regulatory network of hub-shared genes were drawn using the Cytoscape software.

### ScRNA-seq data processing

After sorting the scRNA-seq data, the Seurat package was used to convert the gene expression matrix into Seurat objects and standardize the data. The inclusion criteria for the cells analyzed were as follows: cells with 400–6000 unique molecular identifiers, < 15% of mitochondrial genes, and nCount_RNA > 1000. The data were then integrated using a mutual principal component analysis function. The first 20 principal components were selected to visualize dimensionality reduction using t-distributed stochastic neighbor embedding (t-SNE). Moreover, the Single R package and CellMarker database^10^ were used for cell annotation.

### Drug screening and molecular docking

Based on the hub-shared genes in COVID-19 and silicosis, our study predicted the US Food and Drug Administration (FDA)-approved drugs and experimental compounds included in the Drug SIGnatures DataBase (DSigDB).^11^The screening criterion for potential therapeutic drugs was an adjusted *p*-value < 0.05. To analyze the binding affinities and modes of interaction between the drug candidates and their targets, AutodockVina 1.2.2, an in-silico protein–ligand docking software, was used. The molecular structures of potential therapeutic drugs were retrieved from the PubChem database.^12^The three-dimensional (3D) coordinates of proteins corresponding to hub-shared genes were downloaded from the Protein Data Bank (PDB) database.^13^All protein and molecular files were converted into PDBQT format, excluding all water molecules for docking analysis, and polar hydrogen atoms were included. Finally, PyMol software was used to describe the molecular docking results.

## Results

### Identification of module genes in silicosis and COVID-19

**Fig. 2:**
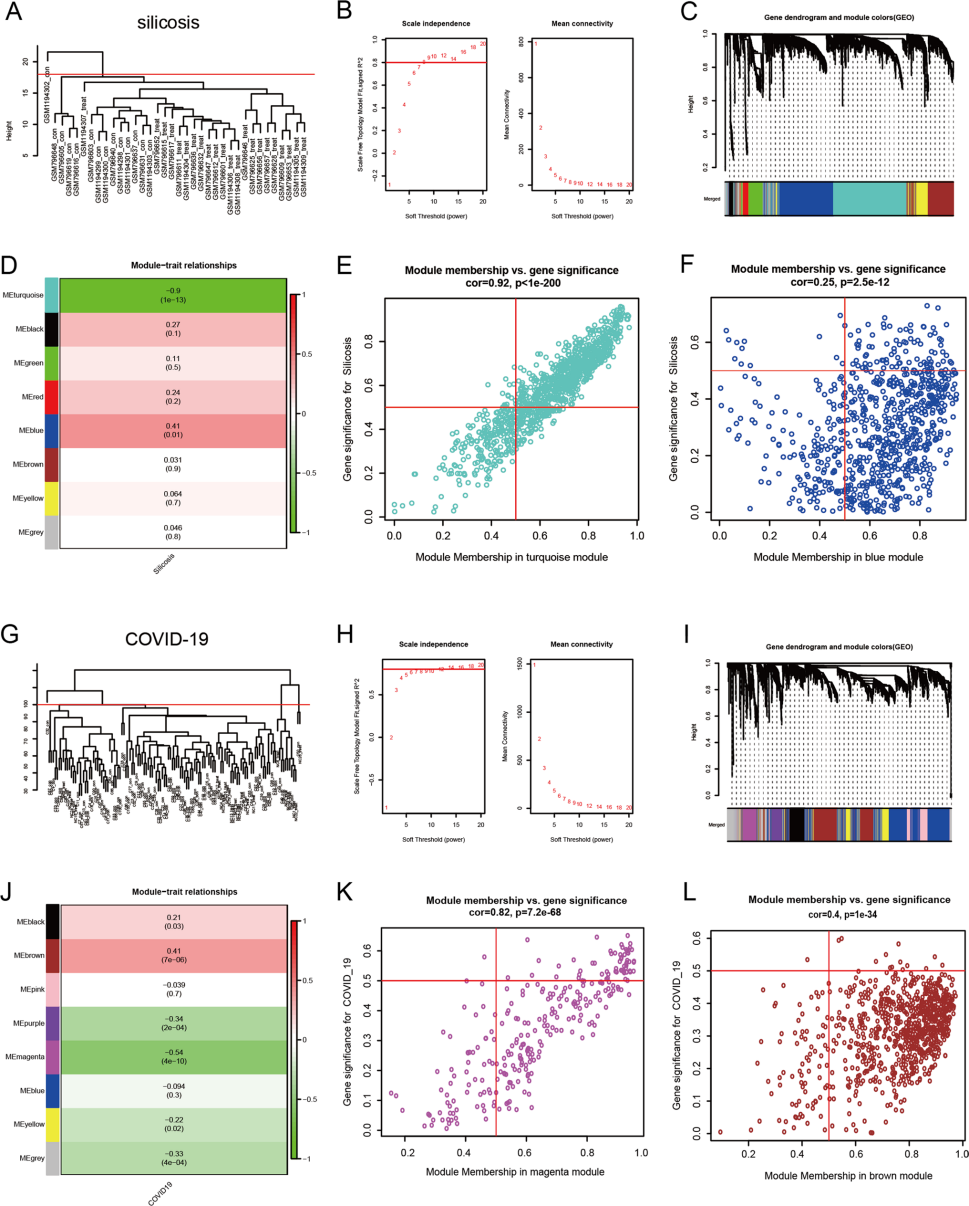
Weighted gene co-expression network analysis of silicosis and COVID-19. **A**, **G** Outlier removal of all the samples; the red line represents lower- and upper boundaries of outlier removal. **B**, **H** The scale-free fitted curve based on R2 according to the soft threshold. **C**, **I** The cluster dendrogram of gene modules with similar expression patterns. **D**, **J** The correlation of modules–trait for disease occurrence. **E**, **F**, **K**, **L** Correlation between module membership and disease gene significance based on scatterplots

For the GSE32147 and GSE49144 datasets, after removing the outlier sample with *h* > 18 (Fig. 2A), the optimal soft threshold, β = 9, was determined according to a scale-free fitting index of 0.80 (Fig. 2B). The silicosis gene set was then divided into eight gene modules, of which the turquoise module had the strongest negative correlation with silicosis (*r* =  − 0.90). Conversely, the blue module had the strongest positive correlation with silicosis (*r* = 0.41) (Fig. 2C, D). Moreover, the correlation between turquoise and blue module membership and the gene significance for silicosis was 0.92 and 0.25, respectively, which is statistically significant (Fig. 2E, F).

For the GSE157103 dataset, after removing the outlier sample with *h* > 100 (Fig. 2G), the optimal soft threshold, β = 12, was determined according to a scale-free fitting index of 0.80 (Fig. 2H). The COVID-19 gene set was then divided into eight gene modules, of which the magenta module had the strongest negative correlation (*r* =  − 0.54) and the brown module had the strongest positive correlation with COVID-19 (*r* = 0.41) (Fig. 2I, J). Moreover, the correlation between magenta and brown module membership and the gene significance for COVID-19 was 0.82 and 0.40, respectively, which is statistically significant (Fig. 2K, L).

### GO pathway enrichment analysis

**Fig. 3:**
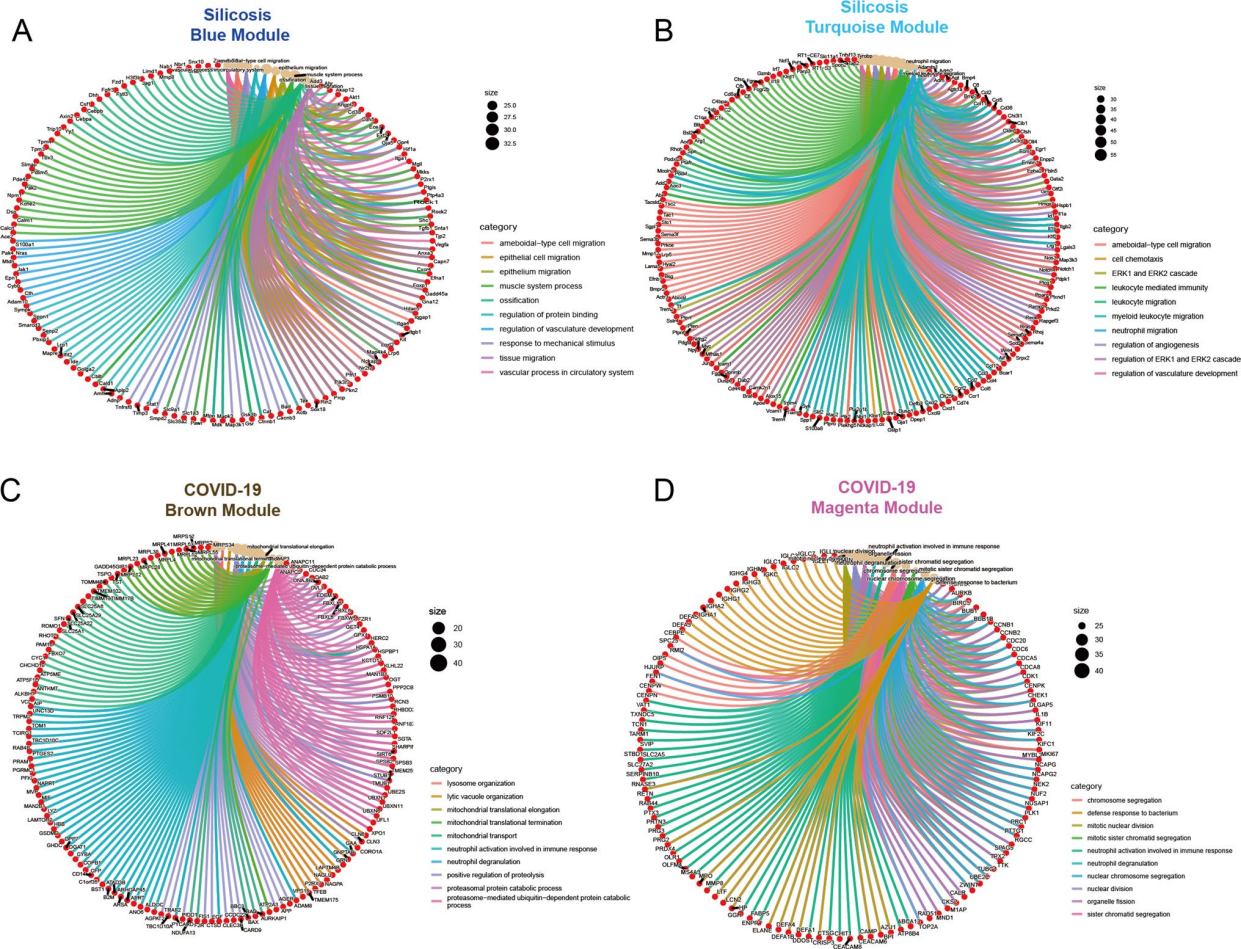
The top 10 most significantly GO-enriched pathways in each module most correlate with silicosis and COVID-19. **A** The top ten significant terms of the blue module (positive) in silicosis. **B** The top ten significant terms of the turquoise module (negative) in silicosis. **C** The top ten significant terms of the brown module (positive) in COVID-19. **D** The top ten significant terms of the magenta module (negative) in COVID-19

For GO enrichment analysis, the top 10 significant terms showed that the blue module of silicosis was mainly involved in ameboidal type cell migration, epithelial cell migration, epithelial migration, muscle system processes, ossification, regulation of protein binding, regulation of vasculature development, response to mechanical stimulus, tissue migration, and vascular processes in the circulatory system (Fig. 3A). Concerning the GO enrichment analysis inside the turquoise module of silicosis, the top 10 significant terms were ameboidal type cell migration, cell chemotaxis, extracellular signal-regulated kinase 1/2 (ERK1 and ERK2) cascade, leukocyte-mediated immunity, leukocyte migration, myeloid leukocyte migration, neutrophil migration, regulation of angiogenesis, regulation of ERK1 and ERK2 cascade, and regulation of vasculature development (Fig. 3B).

The top 10 GO enrichment terms of the brown module in COVID-19 were lysosome organization, lytic vacuole organization, mitochondrial translational elongation, mitochondrial translational termination, mitochondrial transport, neutrophil activation involved in the immune response, neutrophil degranulation, positive regulation of proteolysis, proteasomal protein catabolic process, and proteasome-mediated ubiquitin-dependent protein catabolic process (Fig. 3C). The top 10 GO enrichment terms of the magenta module in COVID-19 were mainly involved in chromosome segregation, defense response to bacteria, mitotic nuclear division, mitotic sister chromatid segregation, neutrophil activation implicated in the immune response, neutrophil degranulation, nuclear chromosome segregation, nuclear division, organelle fission, and sister chromatid segregation (Fig. 3D).

### Identification of shared genes in silicosis and COVID-19

**Fig. 4:**
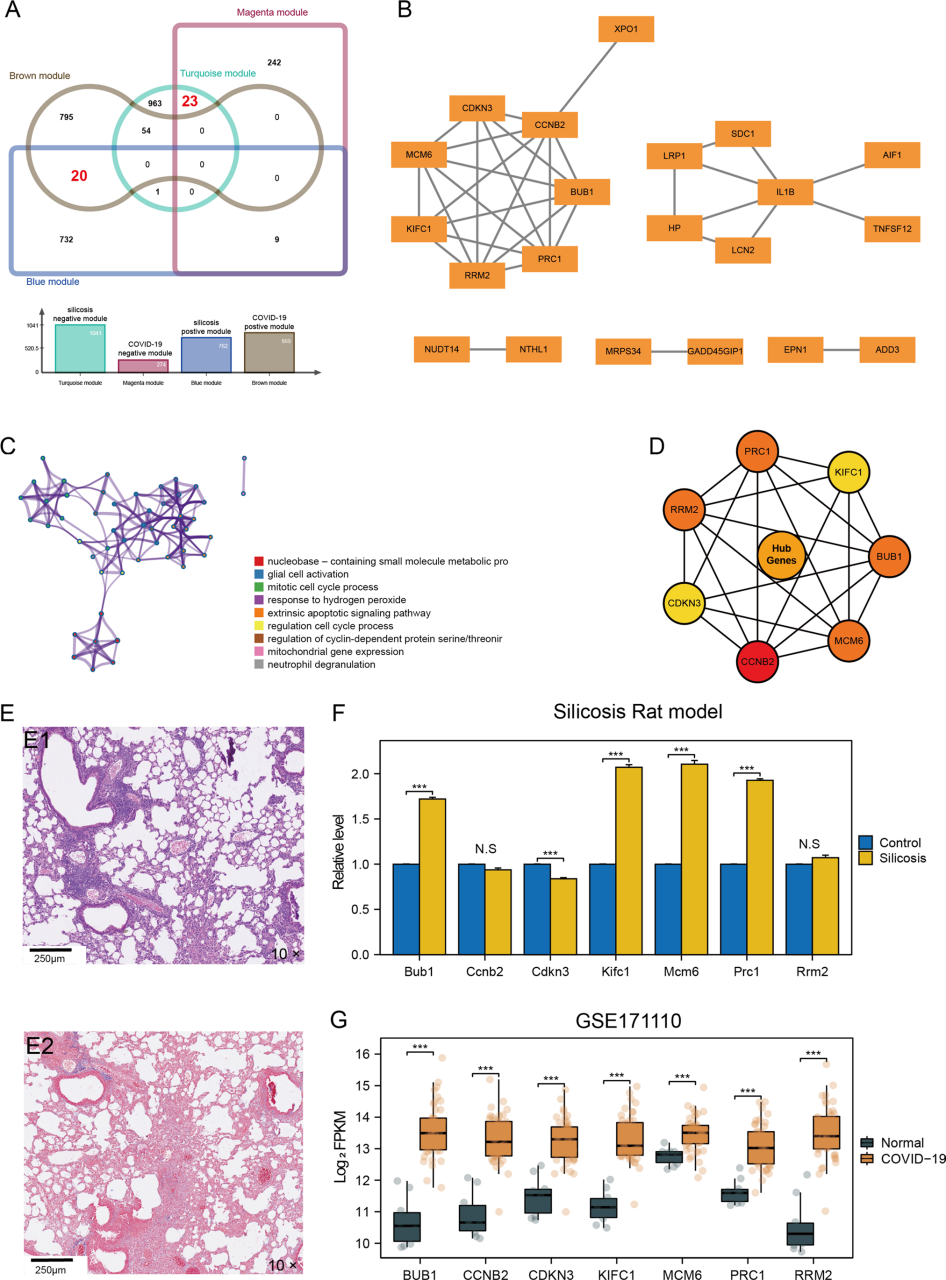
Validation and functional enrichment of shared genes in silicosis and COVID-19. **A** Venn diagram of module genes that are most significantly positively or negatively correlated with disease. **B** PPI network of shared genes. **C** The functional enrichment analysis of the shared genes. **D** Hub-shared genes were filtered in PPI network via the MCODE plugin. **E** Masson trichrome (E1) and HE (E2) staining of rat lung tissue from the silicosis model and normal tissue. **F** RT-qPCR was conducted to validate the expression of hub-shared genes in the rat silicosis model. **G** The COVID-19 validation of hub-shared genes was performed using the GSE171110 dataset. PPI: Protein–protein interaction

According to the Venn diagram, the positive correlation module of silicosis and COVID-19 shared 20 genes, and the negative correlation module shared 23 genes (Fig. 4A). The PPI network of shared genes included 43 nodes and 33 edges, for which the PPI enrichment *p*-value was 1.65 × 10^−05^. After removing the isolated genes, 21 genes were visualized using Cytoscape software (Fig. 4B). Based on the enrichment analysis results, these genes were mainly involved in nucleobase-containing small molecule metabolic processes: glial cell activation, mitotic cell cycle process, response to hydrogen peroxide, extrinsic apoptotic signaling pathway, regulation of cell cycle process, regulation of cyclin-dependent protein serine/threonine, mitochondrial gene expression, and neutrophil degranulation (Fig. 4C). Finally, submodule analysis showed that PRC1, KIFC1, BUB1, MCM6, CCNB2, CDKN3, and RRM2, which were hub-shared genes of silicosis and COVID-19, possessed the highest mCODE scores (Fig. 4D).

We validated the shared genes in silicosis and COVID-19 using animal models and other transcriptome data, respectively. HE and Masson trichrome staining confirmed the successful construction of the rat silicosis model (Fig. 4E). In the rat model exposed to silica inhalation for 4 weeks, Bub1, Kifc1, Mcm6, and Prc1 were upregulated in the silicosis group, while Cdkn3 was downregulated (Fig. 4F). In the peripheral blood bulk RNA sequencing dataset of COVID-19, GSE171110, PRC1, KIFC1, BUB1, MCM6, CCNB2, CDKN3, and RRM2 genes showed statistically significant differences and were upregulated in the COVID-19 group (Fig. 4G).

### TFs, miRNAs, and the disease regulatory network

**Fig. 5:**
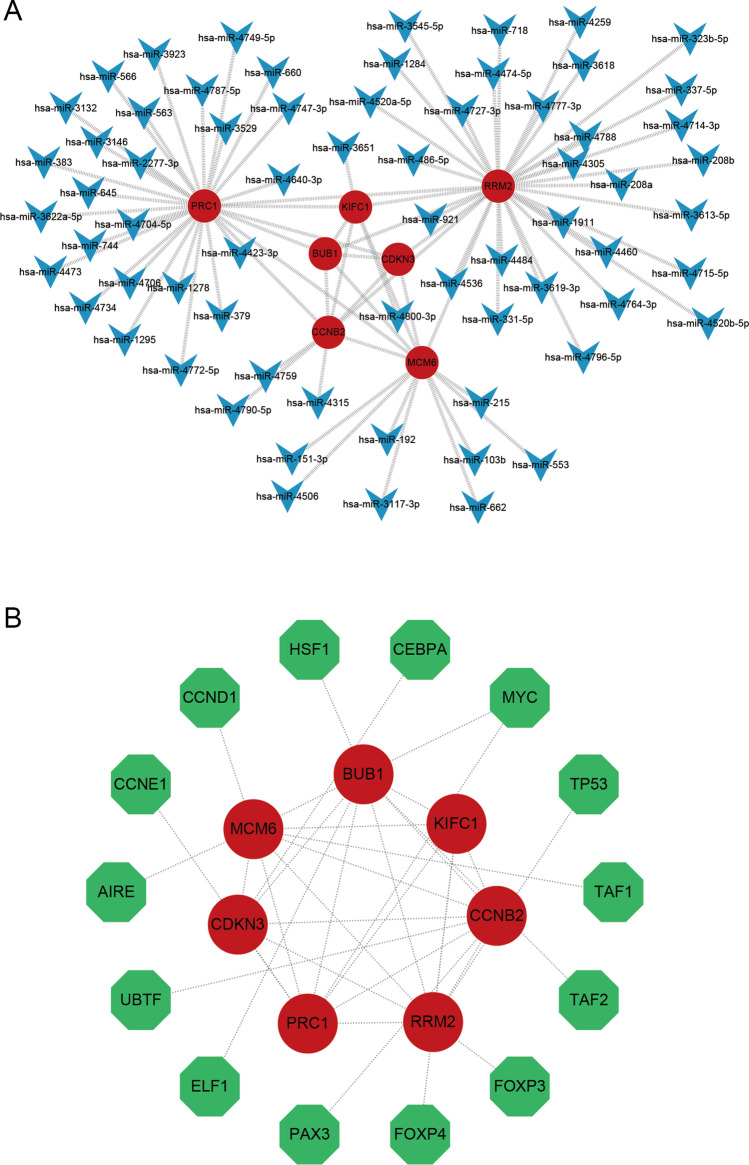
The miRNA-TFs-target regulatory genes network. **A** The miRNA-target genes regulatory network. **B** The regulatory network of TFs and target genes. TFs: transcription factors

**Fig. 6:**
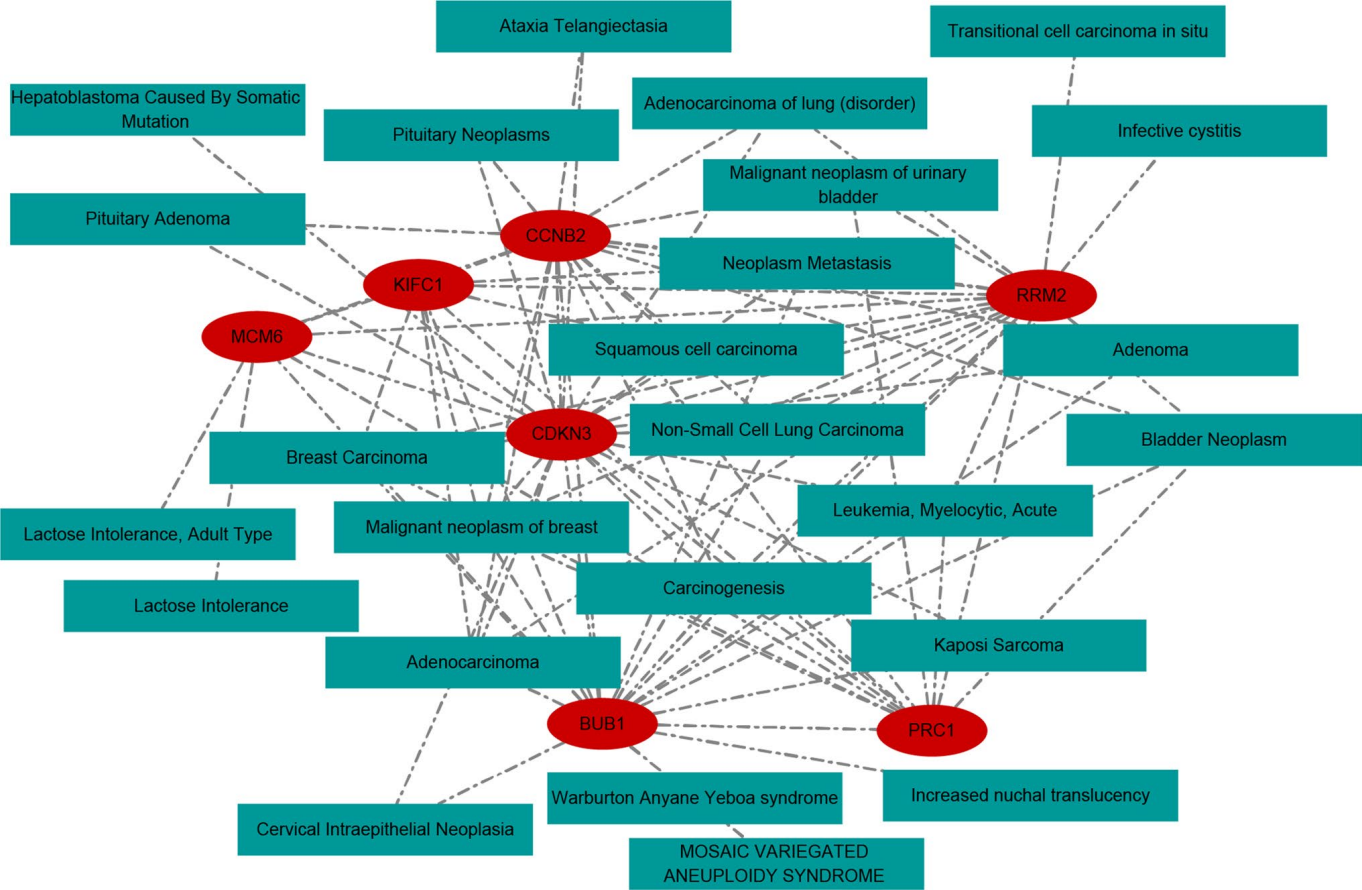
The disease enrichment analysis of shared hub genes

To reveal the regulatory mechanisms of shared genes at the transcriptome level, we utilized a network-based approach to determine the regulatory relationships between TFs and miRNAs. In the regulatory network of miRNAs, these seven shared genes were linked to 67 miRNAs, with RRM2 having the highest number of miRNA linkages (29) (Fig. 5A). Simultaneously, in the TF regulatory network, these shared genes were linked to 11 TFs, including transcription-associated factor 1 (TAF1), forkhead box P3 (FOXP3), paired box 3 (PAX3), E74 like ETS transcription factor 1 (ELF1), upstream binding transcription factor (UBTF), autoimmune regulator (AIRE), cyclins E1 and D1 (CCNE1, CCND1), heat shock transcription factor 1 (HSF1), CCAAT enhancer-binding protein alpha (CEBPA), and MYC (Fig. 5B). The fact that a few genes are shared between different diseases provides a direction for identifying common mechanisms for these diseases. According to the gene-disease association, 25 diseases may share these seven hub genes with silicosis and COVID-19 (Fig. 6). Additionally, we observed that lung diseases, including non-small cell lung carcinoma and lung adenocarcinoma, share these hub genes.

### Immune infiltration analysis of COVID-19

**Fig. 7:**
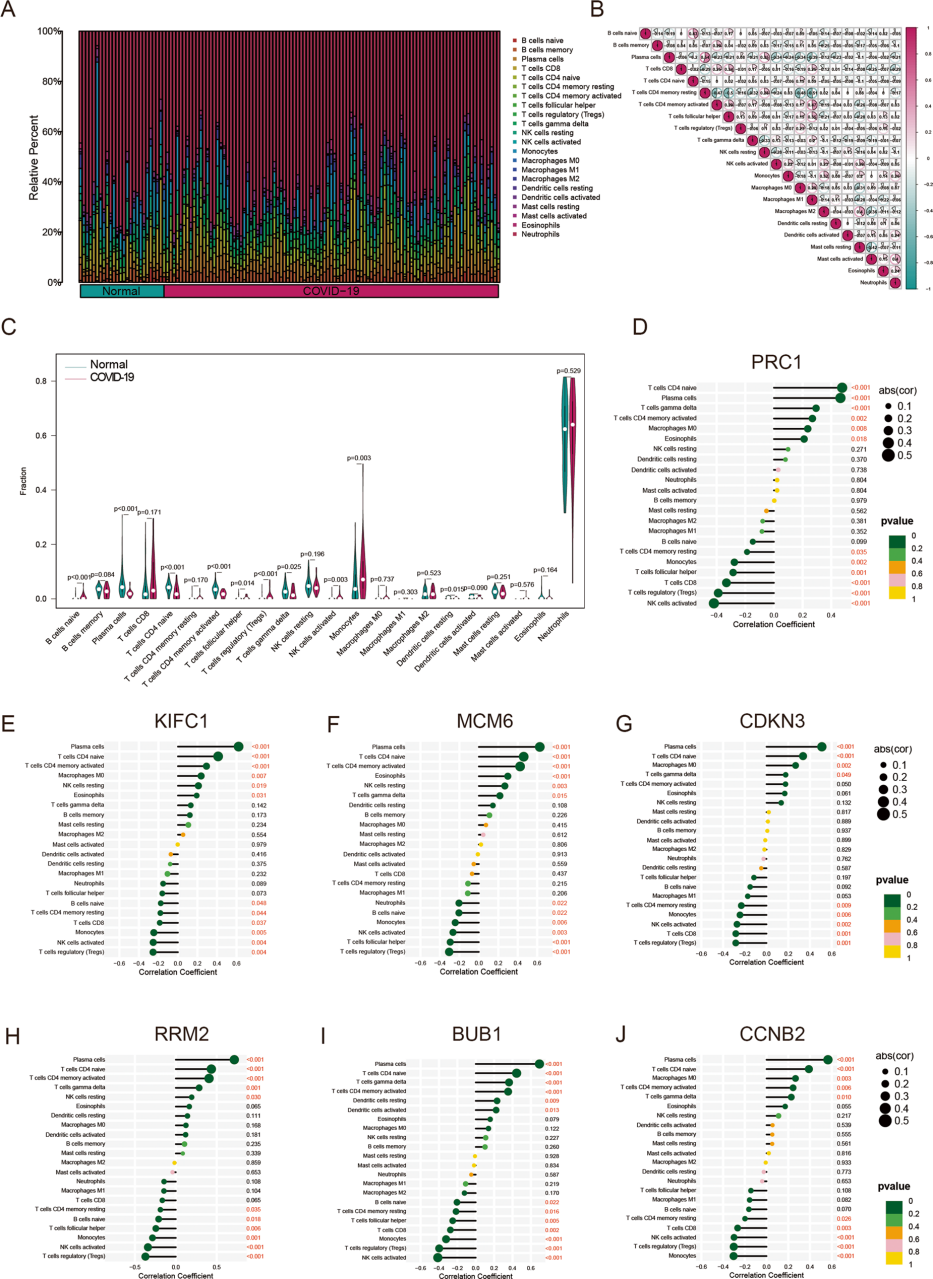
The immune infiltration analysis of COVID-19. **A** The immune infiltration ratio of 22 immune cell types in GSE157103 dataset. **B** Correlation analysis of immune cells. **C** The differential expression analysis of immune cells in normal and COVID-19 patients by CIBERSORT. **D** Lollipop plot of the correlation between seven hub-shared genes (BUB1, PRC1, KIFC1, RPM2, CDKN3, CCNB2, and MCM6) and immune cells

According to the CIBERSORT algorithm, the immune infiltration abundances of 22 immune cells in 128 samples of GSE157103 were evaluated (Fig. 7A). In the correlation analysis of immune cells, resting CD4 + T cells showed a strong positive correlation (*r* = 0.54) with plasma cells. In contrast, resting CD4 + T cells showed a strong negative correlation (*r* =  − 0.51) with M2 macrophages (Fig. 7B). The differences in immune cell abundance between patients with COVID-19 and healthy individuals were then explored. As shown in Fig. 7C, the number of naïve B cells, regulatory T cells, activated natural killer (NK) cells, and monocytes was higher in the COVID-19 group than in the normal group. Moreover, the numbers of plasma cells, naïve CD4 T cells, CD4 memory activated T cells, follicular helper T cells, gamma delta T cells, resting dendritic cells, and activated dendritic cells were higher in the normal group than in the COVID-19 group.

We analyzed the correlation between the seven hub-shared genes and 22 immune cells (Fig. 7D–J). PRC1 had the strongest positive correlation with naïve CD4 T cells and the strongest negative correlation with activated NK cells. KIFC1, MCM6, CDKN3, and RRM2 showed the strongest positive correlations with plasma cells and negative correlations with regulatory T cells. BUB1 showed the strongest positive correlation with plasma cells and the strongest negative correlation with activated NK cells, while CCNB2 had the strongest positive correlation with plasma cells and the strongest positive correlation with monocytes.

### Analysis of COVID-19 single-cell data

**Fig. 8:**
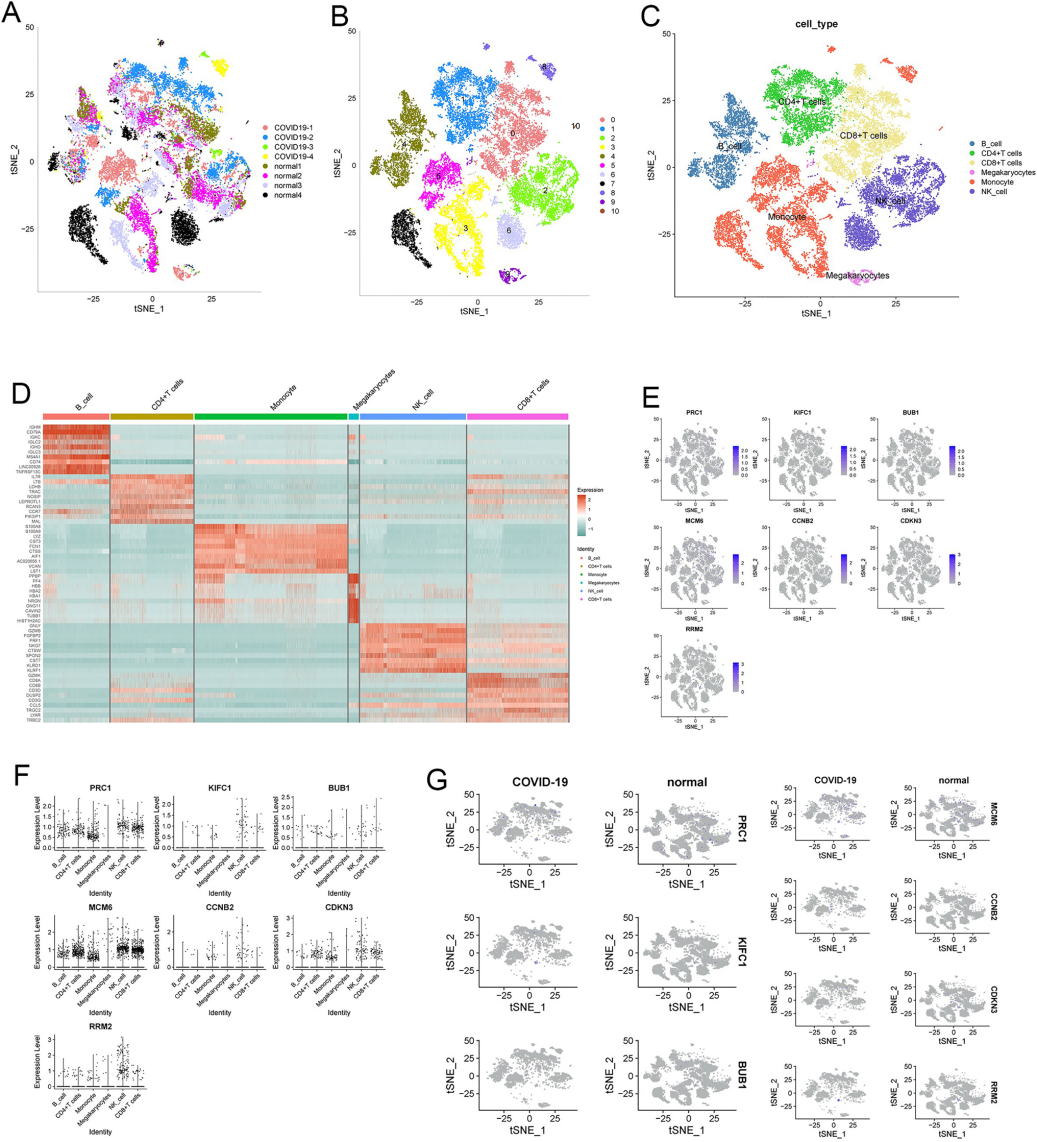
Characterization of hub shared genes cellular localization via single-cell RNA sequencing in GSE182123 dataset. **A** Single-cell subgroups are distributed across different samples. **B** The t-SNE map of 11 clusters. **C** The t-SNE plot of single-cell subpopulation annotation (B cells, CD4 + T cells, megakaryocytes, monocytes, and NK cells). **D** The heatmap of top 10 genes for single-cell subpopulations. **E**, **G** The t-SNE plots of seven hub-shared genes (BUB1, PRC1, KIFC1, RPM2, CDKN3, CCNB2, and MCM6) in each cell subpopulation. **F** The violin plots of seven hubs shared genes in each cell subpopulation. t-SNE: t-distributed stochastic neighbor embedding

Based on the screening criteria, 9115 and 16,316 cells were retained in the COVID-19 and normal groups, respectively (Fig. 8A). After performing t-SNE dimension reduction, 11 cell clusters were identified (Fig. 8B). Combined with manual and single R package annotations, six cell clusters were annotated: B cells, CD4 + T cells, megakaryocytes, monocytes, and NK cells (Fig. 8C). A heatmap of the top 10 genes expressed in each cell cluster is shown in Fig. 8D. In addition, we visualized the cell expression of the seven shared genes on the t-SNE map (Fig. 8E). PRC1 and MCM6 had high expression levels in the various cell subsets, except in megakaryocytes (Fig. 8F). Meanwhile, KIFC1, CCNB2, CKKN3, and RRM2 were expressed at relatively high levels in NK cells. Finally, we visualized the expression levels of the seven genes in the COVID-19 and normal groups using the t-SNE map (Fig. 8G).

### Drug prediction and molecular docking

**Fig. 9:**
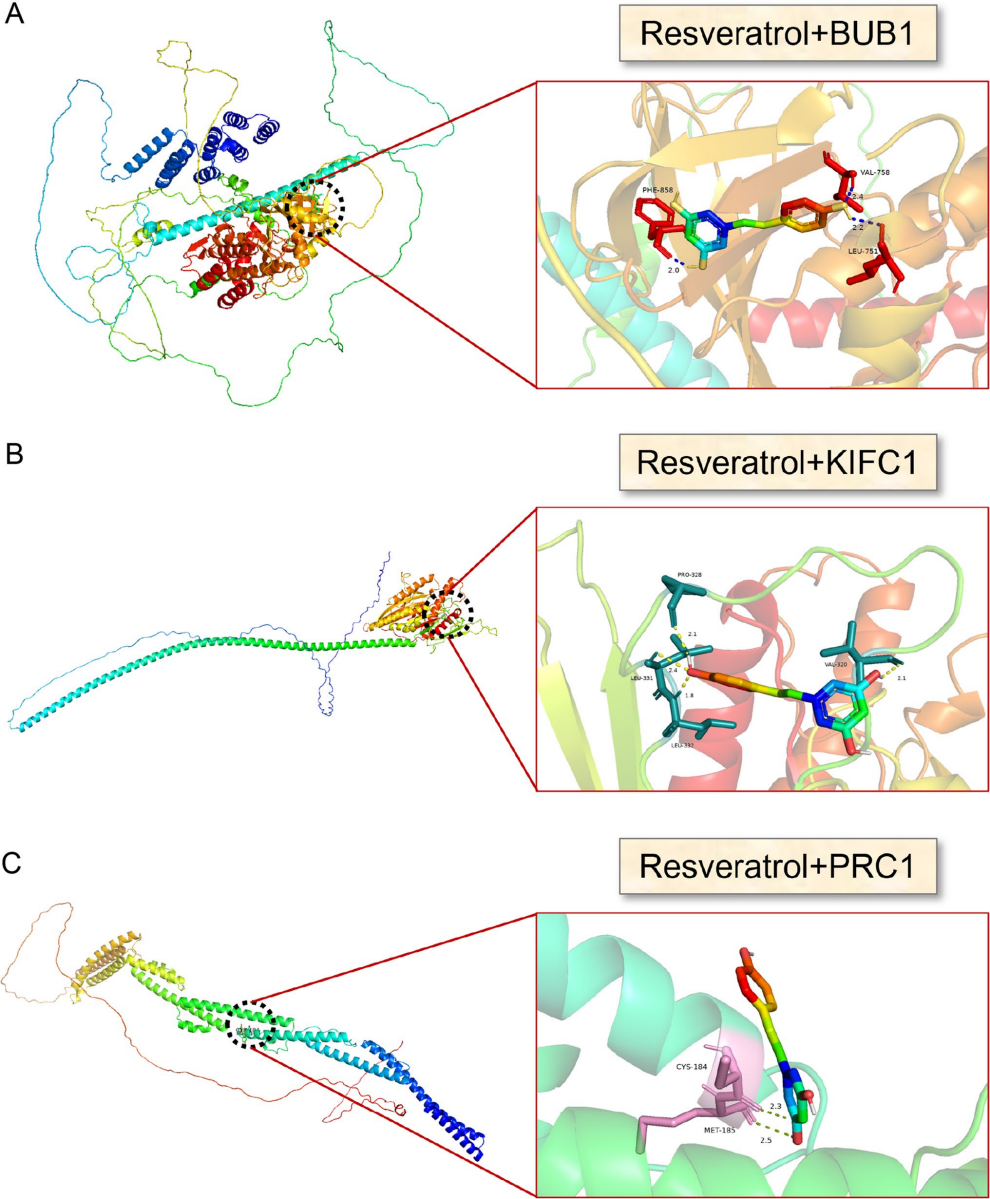
Molecular docking simulation of **A** BUB1, **B** KIFC1, and **C** PRC1 proteins with resveratrol

We predicted small-molecule compounds that might be bound by the seven shared genes in the DSigDB database. As shown in Table 1, the top 10 drugs that may bind to shared genes in the order of their binding score are testosterone, calcitriol, estradiol, cyclosporin A, lucanthone, cryptolepine, dasatinib, piroxicam, troglitazone, and resveratrol. Resveratrol is a natural polyphenol with significant antiviral and antioxidant effects that improve the prognosis of patients with COVID-19 (Ahmadian et al. 2021; Xiao et al. 2021). Therefore, we docked resveratrol with the BUB1, KIFC1, and PRC1 proteins, and it showed suitable binding energies (≤ − 5.0 kcal/mol) (Fig. 9A–C).

**Table 1:** The top 10 drugs predicted by DSigDB database that may bind to shared genes

Drug	Adj. *p*-value	Combined score	Binding genes
Testosterone	< 0.0001	2,551,157.00	CCNB2;RRM2;PRC1;KIFC1;MCM6;BUB1;CDKN3
Calcitriol	< 0.0001	2,055,055.00	CCNB2;RRM2;PRC1;KIFC1;MCM6;BUB1;CDKN3
Estradiol	0.0003	1,173,564.00	CCNB2;RRM2;PRC1;KIFC1;MCM6;BUB1;CDKN3
Cyclosporin A	0.0005	1,057,437.00	CCNB2;RRM2;PRC1;KIFC1;MCM6;BUB1;CDKN3
Lucanthone	< 0.0001	14,473.00	CCNB2;RRM2;PRC1;KIFC1;BUB1;CDKN3
Cryptolepine	< 0.0001	11,319.00	MCM6;BUB1;CDKN3
Dasatinib	< 0.0001	4755.00	RRM2;PRC1;KIFC1;MCM6;BUB1;CDKN3
Piroxicam	< 0.0001	4219.00	CCNB2;RRM2;PRC1;MCM6;BUB1;CDKN3
Troglitazone	< 0.0001	3350.00	CCNB2;RRM2;PRC1;MCM6;BUB1;CDKN3
Resveratrol	< 0.0001	1811.00	PRC1;KIFC1;BUB1

## Discussion

There are multiple variants of COVID-19, which can cause clinical symptoms in various systems, including respiratory, cardiovascular, neurological, gastrointestinal, and dermatological, and many patients with COVID-19 are in critical and even life-threatening conditions (Sharma et al. 2021). Since the start of the COVID-19 pandemic, there have been warnings that patients with preexisting lung conditions are more susceptible to COVID-19 infections. This may be attributed to the damaged immune system and their state of health (Dhikav 2022). In addition, the co-occurrence of COVID-19 and silicosis has been sporadically reported (Naidoo and Jeebhay 2021). However, their shared gene signatures and related biological processes are still unclear. Therefore, this study aimed to identify the common mechanisms of COVID-19 and silicosis using integrated bioinformatics-based methods.

We first performed WGCNA to identify co-expression gene modules that were significantly positively or negatively correlated with COVID-19 and silicosis. We systematically explored GO and KEGG functional enrichment analyses for four co-expressed gene modules. Thereafter, a Venn diagram was drawn based on the lists of genes significantly positively or negatively correlated with the two diseases. Furthermore, we constructed PPI networks and explored the biological pathways of 43 intersecting genes. Seven hub-shared genes representing the intersection genes of the two diseases were identified from the PPI network and used to further construct TF-gene interactions and gene-miRNA regulatory networks. In addition, bulk RNA-seq analysis of COVID-19 was performed to explore the correlation between shared hub genes and immune cells. Based on a single-cell sequencing dataset for COVID-19, the expression of seven key genes was identified in multiple cell clusters. Finally, it was predicted that the antioxidant resveratrol could accurately and directly bind to the three proteins corresponding to the key genes BUB1, KIFC1, and PRC1.

Enrichment analysis revealed that the co-expression gene module was significantly related to silicosis and was mainly enriched in pathways associated with cell migration and inflammation, such as ameboidal-type cell migration, epithelial cell migration, leukocyte migration, and neutrophil migration. These results are consistent with silicosis, which is characterized by inflammatory cell infiltration and fibroblast migration. Similar findings were observed in the co-expressed gene modules that were significantly associated with COVID-19. The results showed that significant module genes were mainly enriched in inflammation and the cell cycle pathways, such as neutrophil activation involved in the immune response, mitochondrial transport, and mitotic nuclear division. Many studies have linked the progression of COVID-19 to oxidative stress. Therefore, mitochondria play an important role in the oxidative homeostasis of cells (Saleh et al. 2020; Singh et al. 2020; Valdés-Aguayo et al. 2021). Moreover, mitochondrial dysfunction is attributed to a heightened inflammatory or oxidative state (Kloc et al. 2020). Our results are consistent with those of the previous studies.

In our study, seven genes (BUB1, PRC1, KIFC1, RRM2, CDKN3, CCNB2, and MCM6) were involved in the interaction between COVID-19 and silicosis. The biological processes behind the role of key genes were clarified. BUB1 performs important functions during mitosis (Bolanos-Garcia and Blundell 2011; Kim and Gartner 2021; Singh et al. 2021). A few studies have demonstrated that BUB1 critically influences the pathophysiological processes of COVID-19 (Agrawal et al. 2022; Jin et al. 2022). In our study, BUB1 negatively correlated with COVID-19 and silicosis, indicating a better prognosis. In addition, the function of CCNB2 in the cell cycle’s regulatory machinery is crucial (Gao et al. 2018). CCNB2 is a vital hub-shared gene in both silicosis and COVID-19 and is a critical hub-shared gene of COVID-19 and lung adenocarcinoma (Yang et al. 2022). We suggested that the expression of these hub genes is the critical factor in the severity of COVID-19 and silicosis, which influence the pathogenesis and clinical feature. Furthermore, these genes are also common diagnostic markers and therapeutic targets for COVID-19 and silicosis.

Resveratrol is a natural polyphenol with antioxidant, anti-inflammatory, heart-protective, and anti-cancer properties. Angiotensin-converting enzyme-2 (ACE2), mainly expressed in endothelial cells, is a cell surface receptor that allows SARS-CoV-2 to enter cells. Multiple studies have revealed that resveratrol exerts its anti-COVID-19 and curative effects by reducing the expression of the ACE-2 receptor and alleviating oxidative stress (de Souza Andrade et al. 2022; Domi et al. 2022; Horne and Vohl 2020; van Brummelen and van Brummelen 2022). Silica-induced silicosis is characterized by progressive lung fibrosis, and previous studies have reported that resveratrol can inhibit pulmonary fibrosis (Chelladurai et al. 2021; Wang et al. 2018, 2021). Our molecular docking results showed that resveratrol could potentially treat COVID-19 and silicosis, which is consistent with the aforementioned literature.

This study has certain inherent limitations. The mechanism by which hub-shared genes regulate the biological processes of silicosis and COVID-19 should be verified experimentally. Meanwhile, due to the lack of scRNA-seq data on silicosis, the cellular localization of hub-shared genes in silicosis needs to be further evaluated. In addition, although molecular docking is a feasible method to predict drugs, it has not yet been verified if resveratrol is effective in the comorbidity of silicosis and COVID-19.

## Conclusion

Using bulk RNA-seq, we identified shared gene clusters that might be involved in the progression of silicosis and COVID-19. The prediction of resveratrol and the construction of regulatory networks for hub-shared genes may provide new insights into managing and treating patients with COVID-19 and silicosis.

## Supplementary Information

Below is the link to the electronic supplementary material.

Supplementary file1 (XLSX 12 KB)

Supplementary file2 (XLSX 10 KB)

## Data Availability

The results, data, and figures in this manuscript have not been published elsewhere, nor are they under consideration by another publisher. All of the material is owned by the authors, and no permissions are required.

## Footnotes

1 https://covid19.who.int

2 http://www.ncbi.nlm.nih.gov/geo/

3 http://www.ehbio.com/test/venn/

4 http://metscape.ncibi.org

5 http://www.bioinformatics.com.cn/

6 https://string-db.org/

7 https://cibersort.stanford.edu/

8 https://maayanlab.cloud/Enrichr/

9 https://www.disgenet.org/

10 http://biocc.hrbmu.edu.cn/CellMarker/

11 http://tanlab.ucdenver.edu/DSigDB

12 https://pubchem.ncbi.nlm.nih.gov/

13 http://www.rcsb.org/pdb/home/home.do
