# Correction to: Exploration of the potential common pathogenic mechanisms in COVID‑19 and silicosis by using bioinformatics and system biology

**DOI:** 10.1007/s10142-023-01165-2

**Published:** 2023-07-14

**Authors:** Yunze Tian, Beibei Yu, Yongfeng Zhang, Sanpeng Zhang, Boqiang lv, Shouping Gong, Jianzhong Li

**Affiliations:** 1 Department of Thoracic Surgery, the Second Affiliated Hospital of Xi’an Jiao Tong University, Xi’an, 710004 Shaanxi Province China; 2 Department of Neurosurgery, the Second Affiliated Hospital of Xi’an Jiao Tong University, Xi’an, 710004 Shaanxi Province China; 3 Operating room, the Second Affiliated Hospital of Xi’an Jiao Tong University, Xi’an, 710004 Shaanxi Province China

**Correction to: Functional & Integrative Genomics (2023)**

**https://doi.org/10.1007/s10142-023-01092-2**

The article “Exploration of the potential common pathogenic mechanisms in COVID‑19 and silicosis by using bioinformatics and system biology”, written by Yunze Tian, Beibei Yu, Yongfeng Zhang, Sanpeng Zhang, Boqiang lv, Shouping Gong and Jianzhong Li, was originally published Online First without Open Access. After publication in volume 23, issue 3 the author decided to opt for Open Choice and to make the article an Open Access publication. Therefore, the copyright of the article has been changed to © The Author(s) 2023 and the article is forthwith distributed under the terms of the Creative Commons Attribution 4.0 International License, which permits use, sharing, adaptation, distribution and reproduction in any medium or format, as long as you give appropriate credit to the original author(s) and the source, provide a link to the Creative Commons licence, and indicate if changes were made. The images or other third party material in this article are included in the article’s Creative Commons licence, unless indicated otherwise in a credit line to the material. If material is not included in the article’s Creative Commons licence and your intended use is not permitted by statutory regulation or exceeds the permitted use, you will need to obtain permission directly from the copyright holder. To view a copy of this licence, visit http://creativecommons.org/licenses/by/4.0.

The original article has been corrected.

